# Unimodal Tree Size Distributions Possibly Result from Relatively Strong Conservatism in Intermediate Size Classes

**DOI:** 10.1371/journal.pone.0052596

**Published:** 2012-12-31

**Authors:** Yue Bin, Wanhui Ye, Helene C. Muller-Landau, Linfang Wu, Juyu Lian, Honglin Cao

**Affiliations:** 1 Key Laboratory of Vegetation Restoration and Management of Degraded Ecosystems, South China Botanical Garden, Chinese Academy of Sciences, Guangzhou, China; 2 Smithsonian Tropical Research Institute, Panamá, República de Panamá; Utah State University, United States of America

## Abstract

Tree size distributions have long been of interest to ecologists and foresters because they reflect fundamental demographic processes. Previous studies have assumed that size distributions are often associated with population trends or with the degree of shade tolerance. We tested these associations for 31 tree species in a 20 ha plot in a Dinghushan south subtropical forest in China. These species varied widely in growth form and shade-tolerance. We used 2005 and 2010 census data from that plot. We found that 23 species had reversed J shaped size distributions, and eight species had unimodal size distributions in 2005. On average, modal species had lower recruitment rates than reversed J species, while showing no significant difference in mortality rates, per capita population growth rates or shade-tolerance. We compared the observed size distributions with the equilibrium distributions projected from observed size-dependent growth and mortality. We found that observed distributions generally had the same shape as predicted equilibrium distributions in both unimodal and reversed J species, but there were statistically significant, important quantitative differences between observed and projected equilibrium size distributions in most species, suggesting that these populations are not at equilibrium and that this forest is changing over time. Almost all modal species had U-shaped size-dependent mortality and/or growth functions, with turning points of both mortality and growth at intermediate size classes close to the peak in the size distribution. These results show that modal size distributions do not necessarily indicate either population decline or shade-intolerance. Instead, the modal species in our study were characterized by a life history strategy of relatively strong conservatism in an intermediate size class, leading to very low growth and mortality in that size class, and thus to a peak in the size distribution at intermediate sizes.

## Introduction

Tree size distributions are useful indicators of the structure and dynamics of tree populations [1]. Tree size distributions may be associated with both life history [2] and population trends [3], [4]. Thus, they have long been of interest to ecologists and foresters concerned with species coexistence, competition and forest management [4].

Size distributions are considered an important indicator of population dynamics. A reversed J-shaped size distribution has been regarded as a proxy of population growth or dynamic equilibrium while a unimodal distribution, with comparatively fewer juveniles relative to adults, has been taken as evidence of population decline [3], [5], [6]. However, species with a relatively large proportion of intermediate-sized individuals dominate in some old growth forests. For example, *Castanopsis chinensis* is the most dominant species (by Importance Value, which is sum of relative density, relative dominance and relative frequency for a species) in an evergreen subtropical forest in Dinghushan [7] and *Pinus Koraiensis* dominates a temperate forest in Changbaishan [8] – both sites that have been well protected for hundreds of years [7], [8]. This raises the question of whether these species are really declining, or whether in their case unimodal size distributions are not indicative of population decline. A long-term study of 216 species in an old-growth forest in Panama found that size distribution did correlate with population growth, but weakly and only in understory species, not canopy species [5].

Wright et al. suggested that in old-growth forests, size distributions are indicative of life history strategy than of population trends [2]. They argued that seedlings and saplings of light-demanding species are relatively rare because they are ephemeral, either dying quickly if shaded, or growing rapidly into larger size classes if light level remains high. In contrast, the small individuals of the shade tolerant species survive well and grow slowly in deep shade. Consistent with this, they found that a measure of the shape of the size distribution was strongly correlated with sapling growth and mortality [2]. Other research also supports this view of size distributions [5], [9], [10]. This provided a link between size distribution and shade tolerance because species that are light-demanding as juveniles have higher growth and lower survival than those that are shade-tolerant [11], . The association of shade tolerance and size distribution was supported in one other study of 25 tree species in old growth rain forest [14]. However, some other studies have not found support for this proposed relationship: for example, a shade tolerant species in a temperate old growth forest also had a unimodal size distribution [15].

If size distributions reflect shade tolerance or life history strategy more generally, they should be stable in the absence of major disturbance, approaching a stationary size distribution whose form depends on size-dependent growth and mortality rates [16]. Stationary size distributions can always be obtained from size-dependent growth and mortality functions numerically [17], [18], [19], [20]. In some cases, stationary size distributions can be determined analytically [10]. Comparison of the observed size distribution with the predicted stationary distribution provides a test of whether the population is at equilibrium, and whether size distributions can be explained by life history.

In this study, we tested a series of hypotheses regarding interspecific variation in tree size distributions in a Monsoon evergreen broadleaf forest. Specifically, we tested whether modal size distributions are associated with (1) declining per capita population growth rate, (2) shade-intolerance, (3) higher mortality, and (4) lower recruitment. We further evaluated whether observed size distributions could be explained as consequences of observed size-dependent mortality and size-dependent growth, under the assumption of demographic equilibrium. Our findings show that modal size distributions need not reflect either population decline or light-demanding juveniles, but can instead emerge as a consequence of a life history strategy characterized by an intermediate diameter class with relatively low mortality and growth.

## Materials and Methods

### Ethics Statement

No specific permits were required for the described field studies. Our study site (Dinghushan Nature Reserve) is owned by the Chinese government and managed by South China Botanical Garden, Chinese Academy of Sciences. We freely conducted our research in Dinghushan, as permited under the Regulations of the People’s Republic of China on Nature Reserves. Our field studies did not involve endangered or protected species.

### Study Site

The study site was located in Dinghushan nature reserve (112°30′39′′–112°33′41′′E,23°09′21′′–23°11′30′′N) established in 1956. The nature reserve, covering an area of 1155 ha, is characterized by a south subtropical monsoon climate, with a mean annual temperature of 20.9°C and mean annual precipitation of 1929 mm. The vegetation is Monsoon evergreen broadleaved forest with floristic composition which is transitional between the subtropical and tropical. In 2005, a 20 ha forest dynamic plot was established in the core of the nature reserve, which has been undisturbed for over 400 years. According to previous research on the vegetation of that area, the forest is in succession from a community dominated by *Cryptocarya concinna*, *Castanopsis chinensis, Cryptocarya chinensis*, and *Schima superba* to one dominated only by *Cryptocarya concinna* and *Cryptocarya chinensis* [21].

The plot was first censused in 2005 following the standard methods of the Center for Tropical Forest Science [22]. All free-standing woody plants with diameter at breast height (DBH) ≥1 cm were mapped, tagged, and identified to species, and their DBHs were measured with 0.1 cm precision [7]. The first inventory found 71,617 individuals belonging to 210 species, 119 genera and 56 families. The plot was recensused in 2010. During the recensus, DBHs for all live stem were remeasured; deaths were recorded, and new stems with DBH ≥1 cm DBH threshold were recorded as recruits.

Analyses were done for the thirty-one tree species having 500 or more individuals in the 2005 census, henceforth referred to as the focal species. Species were classified into different degrees of shade tolerance, termed categorical shade tolerance, according to expert knowledge on the frequency of occurrence in canopy gaps or closed canopy [23], [24]. If a species tends to occur in gaps, it is classified as light demanding; if in dense canopy, classified as shade tolerant. If the trend is not so obvious, it is classified as intermediate. This classification criterion is fundamentally similar to the “tendency to recruit in gap” used in other studies [25]. Among our focal species, 2 are light-demanding species, 19 intermediate, and 10 shade-tolerant.

We also calculated a continuous measure of shade tolerance, hereafter continuous shade tolerance, from the relationship between survival probability and neighborhood crowding, as crowding is negatively correlated with light availability. We first fit species-specific logistic regression between the status (alive or dead) in recensus and two independent variables: log-transformed neighborhood crowding [26] and log-transformed DBH. We then predicted the juvenile survival probability with the 5^th^ percentile of the DBHs of each species and the 95^th^ percentile of neighborhood crowding of all individuals in that plot based on the logistic fit. This survival probability was taken as the species-specific continuous shade tolerance.

### Analysis

#### Characterizing size distributions

We classified the initial and final observed size distribution of each focal species as unimodal, multimodal, or simply declining (strictly speaking, nonincreasing) based on visual inspection of histograms constructed as described below. We use modal to refer to unimodal and multimodal size distributions combined, and reversed-J to refer to the declining distributions. We first established size class divisions for each species that were useful for assessing size distribution shape, given available sample sizes. We started with even intervals that spanned the range of diameters in the species, and then combined empty intermediate size classes with neighboring size classes until at least one individual was present in each size class. For most species we started with intervals of 1 cm; for species with large numbers of small individuals, such as *Blastus cochinchinensis* and *Cryptocarya concinna*, we started with smaller intervals, such as 0.1 and 0.2 cm. We then counted the number of individuals in each size class, and determined the number of individuals per 1-cm dbh interval by dividing this number by the width of the size class.

We tested whether or not each size distribution was significantly modal. We compared the confidence intervals of the tree densities in the peak size class with the confidence intervals for the tree densities in the neighboring size classes. If non-overlapping size classes were found at both smaller and larger size classes, the peak was considered to be statistically significant.

We further characterized the modal size distributions by the location of their peaks. We evaluated the peak location in 1000 bootstrapped samples in order to calculate 95% confidence intervals on the peak location. The bootstrap was based on data for all 20×20 m quadrats. At each step, we resampled these quadrats with replacement, and determined the peak location in the sampled data. The 1000 bootstrapped samples yielded a distribution for the peak location and the 2.5^th^, 97.5^th^ percentiles were taken as the 95% confidence interval of the peak location.

We measured the rate of decline of the reversed J size distributions by fitting negative exponential functions to the reversed J size distributions. The fitted function took the form
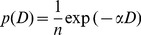
(1)where *D* is diameter, *p(D)* is the probability density at diameter D, 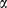 is the fitted rate parameter, and *n* is a normalization constant (not a free parameter). This function was fitted with maximum likelihood following the methods described in great detail in [10]. We fitted this function for 1000 bootstrapped samples in order to obtain 95% confidence intervals on the rate parameter, 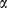. As before, bootstrapping was done over 20×20 m quadrats.

#### Size distributions, demographic rates, and shade-tolerance

We calculated the annual per capita population growth rate, 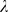, for each species from
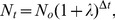
(2)where *N_o_* was the initial population size (here the 2005 population size), *N_t_* the population size at time *t* (here the 2010 population size), and 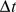 the time that elapsed between two observations of population size, in years (here 5). Note that a growing population will have positive values of 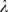, while a shrinking population will have negative values. Confidence intervals on the per capita population growth were computed from 1000 bootstraps over 20×20 m quadrats; these were used to evaluate whether annual per capita population growth rates were significantly different from zero. We tested whether annual per capita population growth rates varied among species depending on the shape of their size distributions (modal vs. reversed J) using the Wilcoxon rank sum test.

We calculated the annual per capita mortality rate, *m*, for each species from
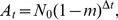
(3)where *A_t_* is the number of survivors at time *t*. We calculated the per capita recruitment rate, *r*, for each species as 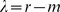. (We didn’t calculate the recruitment rate directly from the number of recruits because the mortality rates of the recruits couldn’t be estimated.) We tested whether mortality and recruitment rates varied among species depending on the shape of their size distributions using Wilcoxon rank sum tests.

We tested for an association among species between the shape of the size distribution and the categorical shade tolerance using a chi-square test. We tested for an association between size distribution and continuous shade tolerance using a two sample t-test.

#### Comparing observed size distributions with equilibrium expectations

We tested whether size distributions were at equilibrium by comparing the observed size distributions with those expected based on observed mean growth and mortality as functions of diameter (eq (4)). The expected equilibrium size distributions were calculated using the equation
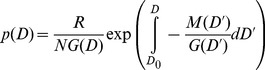
(4)where *R* was the recruitment rate, *N* was the abundance of the focal species at the initial census, *G* and *M* were absolute diameter growth and mortality rate as functions of diameter, respectively, and *D_0_* was the size of individuals upon recruitment [20]. (All rates were calculated per census interval.) Size-dependent growth and mortality functions were fit as a function of log-transformed diameter using LOESS, i.e., locally weighted scatter plot smoothing, using the function loess in R with span 1 (i.e., all points are included) and tri-cubic weighting. To evaluate the confidence in these predictions, we conducted 1000 bootstraps over 20×20 m quadrats and used these to generate 1000 separate predictions for each species. These 1000 bootstrapped predictions were the basis for the assignment of the shape, as well as for calculating confidence intervals on the predicted number of trees in each size class, on the predicted location of the peak in modal species (the location was recorded for each bootstrap), and on fitted negative exponential parameter in reversed J species.

We compared predicted size distributions with observed distributions using multiple tests. (1) We assessed whether the predicted and observed size distributions had the same general shape (modal vs. reversed J). (2) We compared the size distribution parameters of the predicted distributions with those of the observed distributions by examining the peak location for modal species and the negative exponential rate parameter for reversed J species. (2a) We tested if confidence intervals overlapped between predicted and observed parameters for each species, and (2b) we calculated Pearson’s correlation coefficients between predicted and observed parameters over species. (3) We compared log-transformed predicted and observed tree densities in different size classes. For each species, (3a) we quantified the proportion of cases in which the confidence intervals of predicted tree densities overlapped with the confidence intervals of the observed densities, and (3b) we calculated Pearson’s correlation coefficients between predicted and observed densities over size classes.

All data analyses were performed in R 2.13.1 [27].

## Results

### Observed Size Distributions

**Figure 1:**
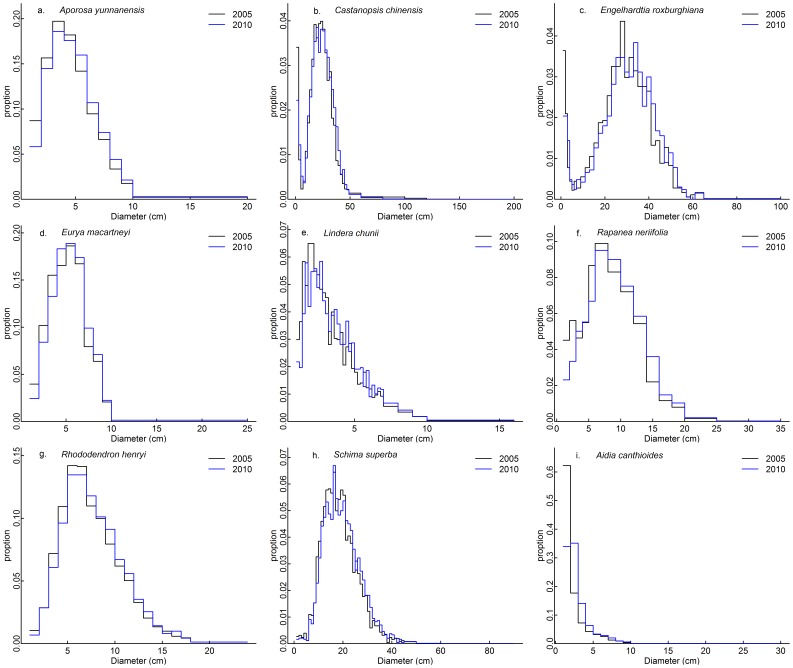
Size distributions of the eight modal species (a-h, all unimodal) and of the most abundant reversed J species (i). Corresponding figures for the remaining 22 reversed J species were given in Figure S1.

Of the 31 species with 500 or more individuals, 8 were classified as modal (Table 1; Figure 1 a–h) and the remaining 23 as reversed J shaped (Table 1; e.g., Figure 1i, Figure S1). Among the 23 reversed J species, five of them have non significant peaks at intermediate sizes in 2005 (Figure S1). Three of these five species (*Blastus cochinchinensis*, *Lindera metcalfiana* and *Sarcosperma laurinum*) became significantly unimodal in 2010 (Table 1; Figure S1). Another reversed J species, *Ilex ficoidea*, also became modal in 2010. The size distributions of the modal species didn’t change much between 2005 and 2010 (Figure 1), although most species had reduced proportions of stems in the smallest size class in the later census (Figure 1).

**Table 1:** The focal species, their abundances, and key statistics on their size distributions in 2005 (05) and 2010 (10).

Species	DST	A05	A10	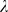 (%)	*r* (%)	*m* (%)	S05	S10	Obs.Par(CI)	Pred.Par(CI)	Pearson’r	Proportion nonsignificantly different
APORYU	ST	4100	3594	−2.59	0.45	3.05	M	M	3.5 (3.0–4.0)	2.5 (2.0–10.0)	0.834**	0.30
CASTCH	I	2840	2882	0.27	1.28	1.01	M	M	24 (15.0–25.0)	6.0 (5.0–7.0)	0.665***	0.20
ENG2RO	LD	909	856	−1.19	0.51	1.71	M	M	28 (25.0–31.0)	4.5 (1.0–5.0)	0.143	0.49
EUR2MA	LD	582	539	−1.52	0.11	1.63	M	M	5.5 (3.0–6.0)	5.5 (1.0–9.0)	0.987***	1.00
LIN1CH	I	2310	2013	−2.69	1.13	3.82	M	M	1.9 (1.4–2.0)	1.1 (1.0–2.0)	0.904***	0.53
RAPANE	I	820	789	−0.77	0.28	1.05	M	M	7 (6.0–8.0)	9.0 (1.0–20.0)	0.992***	0.33
RHO3HE	ST	1710	1629	−0.99	0.18	1.17	M	M	5.5 (5.0–6.0)	5.5 (4.0–6.0)	0.653**	0.39
SCHISU	I	2550	2436	−0.87	0.11	0.99	M	M	16.5 (13.0–20.0)	5.5 (2.0–9.0)	0.42**	0.38
ACMEAC	ST	1980	1968	−0.14	1.24	1.38	rJ	rJ	0.13 (0.12–0.14)	0.07 (0.06–0.07)	0.86***	0.33
AID2CA	ST	6400	8681	6.29	7.40	1.10	rJ	rJ	0.74 (0.70–0.79)	0.18 (0.18–0.19)	−0.163	0.20
ARD2QU	I	4200	3915	−1.39	0.94	2.33	rJ	rJ	1.14 (1.09–1.19)	0.60 (0.53–2.98)	0.91*	0.80
BLASCO	ST	4710	4302	−1.81	1.86	3.67	rJ	rJ	1.59 (1.52–1.67)	0.96 (0.07–6.11)	0.928***	0.77
CAN4DI	ST	624	602	−0.72	1.57	2.28	rJ	rJ	0.24 (0.21–0.27)	0.04 (0.04–0.08)	−0.705***	0.80
CARABR	I	753	1031	6.49	7.52	1.03	rJ	rJ	0.60 (0.52–0.70)	0.21 (0.19–0.24)	0.831*	0.75
CRAIKW	I	3390	3222	−1.01	0.09	1.10	rJ	M	0.13 (0.13–0.14)	0.94(0.94–0.94)	−0.922	0.16
CRY1CH	I	3590	3681	0.51	1.42	0.91	rJ	rJ	0.18 (0.17–0.19)	0.07 (0.06–0.07)	0.954***	0.053
CRY2CO	I	4550	4750	0.86	5.03	4.17	rJ	M	1.15 (1.01–1.32)	0.89 (0.59–1.50)	0.875***	0.41
GAR1OB	LD	661	343	−12.30	0.26	12.55	rJ	rJ	0.26 (0.24–0.29)	1.25 (0.84–1.25)	0.813***	0.17
ILE5FI	ST	651	702	1.52	1.74	0.22	rJ	rJ	0.18 (0.15–0.21)	0.09 (0.06–0.09)	0.918***	0.28
LIN5ME	I	2490	2287	−1.69	1.55	3.25	rJ	M	0.40 (0.37–0.42)	0.55 (0.37–2.33)	0.952***	1.00
MAC2SA	LD	793	806	0.33	3.10	2.77	rJ	rJ	0.41(0.33–0.48)	0.19 (0.19–0.19)	0.73*	0.64
MAC3BR	I	1210	1177	−0.57	1.09	1.66	rJ	rJ	0.19 (0.17–0.21)	0.15 (0.12–0.23)	0.912**	0.65
MAC4CH	I	543	512	−1.17	0.74	1.91	rJ	rJ	0.06 (0.05–0.07)	0.07 (0.04–0.09)	0.696***	0.85
MEM1LI	ST	1360	1383	0.29	1.27	0.97	rJ	rJ	0.43 (0.41–0.46)	0.23 (0.22–0.24)	0.089	0.25
MIC3PE	ST	1350	1408	0.82	3.32	2.50	rJ	rJ	0.85 (0.78–0.94)	0.21 (0.16–0.22)	0.871**	0.20
NEO4UM	I	1480	1222	−3.80	0.48	4.28	rJ	rJ	0.32 (0.29–0.35)	2.33 (1.09–2.50)	0.805***	0.27
ORM2GL	I	2860	2728	−0.93	1.00	1.93	rJ	rJ	0.40 (0.37–0.44)	0.16 (0.10–0.17)	0.947***	0.20
PSYCAS	I	956	681	−6.55	0.64	7.19	rJ	M	1.07 (1.00–1.15)	1.71 (1.46–6.81)	0.892***	1.00
SARCLA	ST	1730	1663	−0.75	0.83	1.58	rJ	M	0.23 (0.22–0.25)	0.11 (0.10–0.11)	0.884***	0.15
SYZ2RE	I	6260	6006	−0.83	0.39	1.22	rJ	rJ	0.28 (0.26–0.29)	0.06 (0.06–0.09)	−0.348	0.61
XANTHA	I	2020	2007	−0.08	0.84	0.92	rJ	rJ	0.27(0.25–0.29)	0.14 (0.10–0.14)	0.88***	0.16

Degree of shade tolerance (DST) is classed as light-demanding (LD), intermediate (I), or shade-tolerant (ST). Abundances (A05 and A10) are given in total number of individuals ≥1 cm DBH on the entire 20-ha plot. Per capita population growth rate (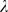), recruitment rate(*r*) and mortality rate (*m*) are given in % per year. The shapes of the size distributions (S05 and S10) are classified as modal (M) or reversed J (rJ). The observed size distribution parameters (peak location for modal species, or rate of decline for reversed J species) as well as those predicted from size-dependent growth and mortality functions are given with 95% CIs from bootstrapping over 20×20 m subplots. Quantitative comparisons of observed size distributions with predicted equilibrium size distributions are pearson correlation coefficient between the predicted and observed log-transformed proportions of individuals in different size classes (Pearson’r) and proportion of size classes of which the predicted tree densities were not significantly different from the observed tree densities (Proportion nonsignificantly different). Significance codes were:

* 0.01≤p<0.05;

** 0.001≤p<0.01;

*** p<0.001.

ACMEAC: *Acmena acuminatissima*; AID2CA: *Aidia canthioides*; APORYU: *Aporosa yunnanensis*; ARD2QU: *Ardisia quinquegona*; BLASCO: *Blastus cochinchinensis*; CAN4DI: *Canthium dicoccum*; CARABR: *Carallia brachiata*; CASTCH: *Castanopsis chinensis*; CRAIKW: *Craibiodendron kwangtungense*; CRY1CH: *Cryptocarya chinensis*; CRY2CO: *Cryptocarya concinna*; ENG2RO: *Engelhardtia roxburghiana*; EUR2MA: *Eurya macartneyi*; GAR1OB: *Garcinia oblongifolia*; ILE5FI: *Ilex ficoidea*; LIN1CH: *Lindera chunii*; LIN5ME: *Lindera metcalfiana*; MAC2SA: *Macaranga sampsoni*; MAC3BR: *Machilus breviflora*; MAC4CH*: Machilus chinensis*; MEM1LI: *Memecylon ligustrifolium*; MIC3PE: *Mischocarpus pentapetalus*; NEO4UM: *Neolitsea umbrosa*; ORM2GL: *Ormosia glaberrima*; PSYCAS: *Psychotria asistica*; RAPANE: *Rapanea neriifolia*; RHO3HE: *Rhododendron henryi*; SARCLA: *Sarcosperma laurinum*; SCHISU: *Schima superba*; SYZ2RE: *Syzygium rehderianum*; XANTHA: *Xanthophyllum hainanense*.

### Size Distributions and Per Capita Population Growth

**Figure 2:**
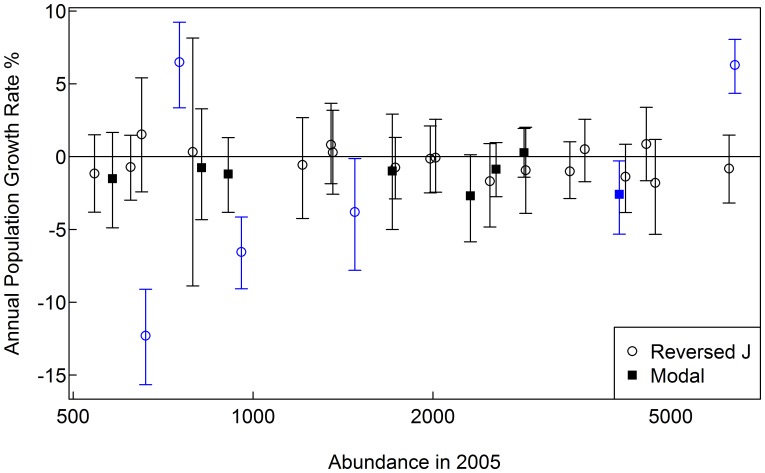
Annual per capita population growth rates of the focal species. Vertical bars denote the 95% confidence interval from 1000 bootstraps over 20×20 quadrats. Blue highlights changes that are significantly different from zero.

Only six species had significant annual per capita population growth rates between 2005 and 2010, with two reversed J species showing significant increases, and three reversed J and one modal species showing significant decreases (Figure 2). Reversed J shaped species and modal species did not differ significantly in their annual per capita population growth rates (Wilcoxon Rank Sum test, W = 125, p-value = 0.1448).

### Size Distributions, Demography, and Shade-tolerance

Modal species had significantly lower recruitment rates than reversed-J species (Wilcoxon rank sum test: W = 149, p-value = 0.00865). There was no significant association between the shape of the size distribution and the mortality rate (W = 107, p-value = 0.5203). There was also no significant association between the shape of the size distribution and either the categorical shade tolerance (X-squared = 0.7901, df = 2, p-value = 0.6737; Table 2) or the continuous shade tolerance (t = 0.4845, df = 22.092, p-value = 0.6328).

**Table 2:** A contingency table between the shape of size distribution and categorical shade tolerance.

Categorical Shade tolerance	Modal	Reversed J
Shade tolerant	2	8
Intermediate	5	14
Light demanding	1	1

The figure in the cells indicated the number of species falling into each category.

### Observed Size Distributions vs. Equilibrium Expectations

Four of the eight modal species were expected to have a significant peak at intermediate sizes, therefore a modal size distribution. Another three species were expected to have a peak at intermediate sizes, but their confidence intervals contained the smallest size class. The remaining modal species was expected to have its peak at the smallest size class, thus a reversed J size distribution. Five of the initial reversed J species were expected to have a non-significant peak at intermediate size: *Ardisia quinquegona*, *Cryptocarya concinna*, *Blastus cochinchinensis*, *Machilus chinensis*, and *Lindera metcalfiana* (Figure S2). Two of these species, *Blastus cochinchinensis* and *Lindera metcalfiana*, had unimodal size distribution in 2010.

**Figure 3:**
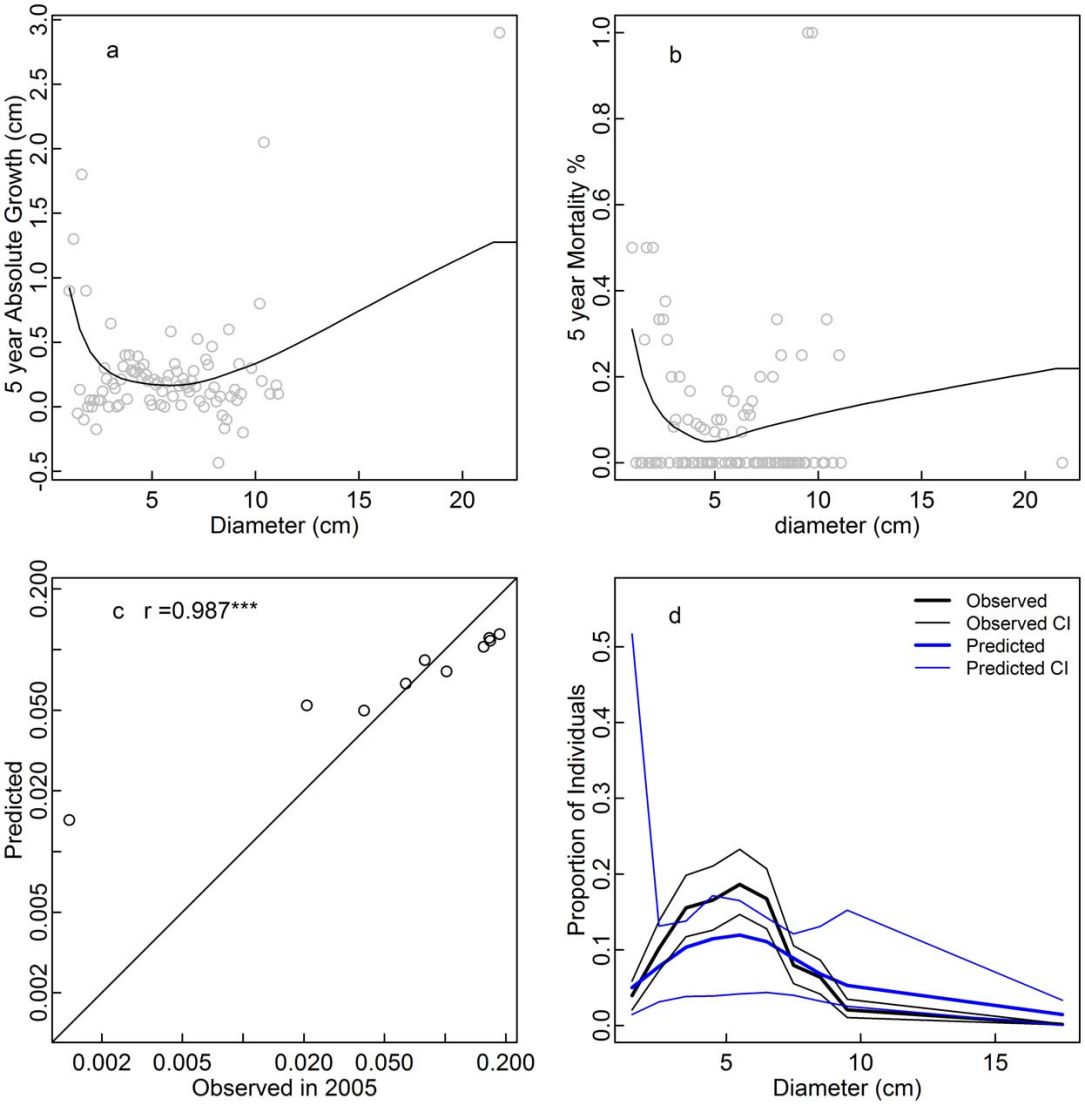
Five year absolute diameter growth (a), five year mortality rate (b), and observed 2005 vs. predicted equilibrium proportions of individuals in each size class (c,d) for *Eurya macartneyi*. The prediction was derived from growth and mortality functions under the assumption that the population is at demographic equilibrium. Confidence intervals were obtained from bootstrapping over 20×20 m subplots (d).

**Figure 4:**
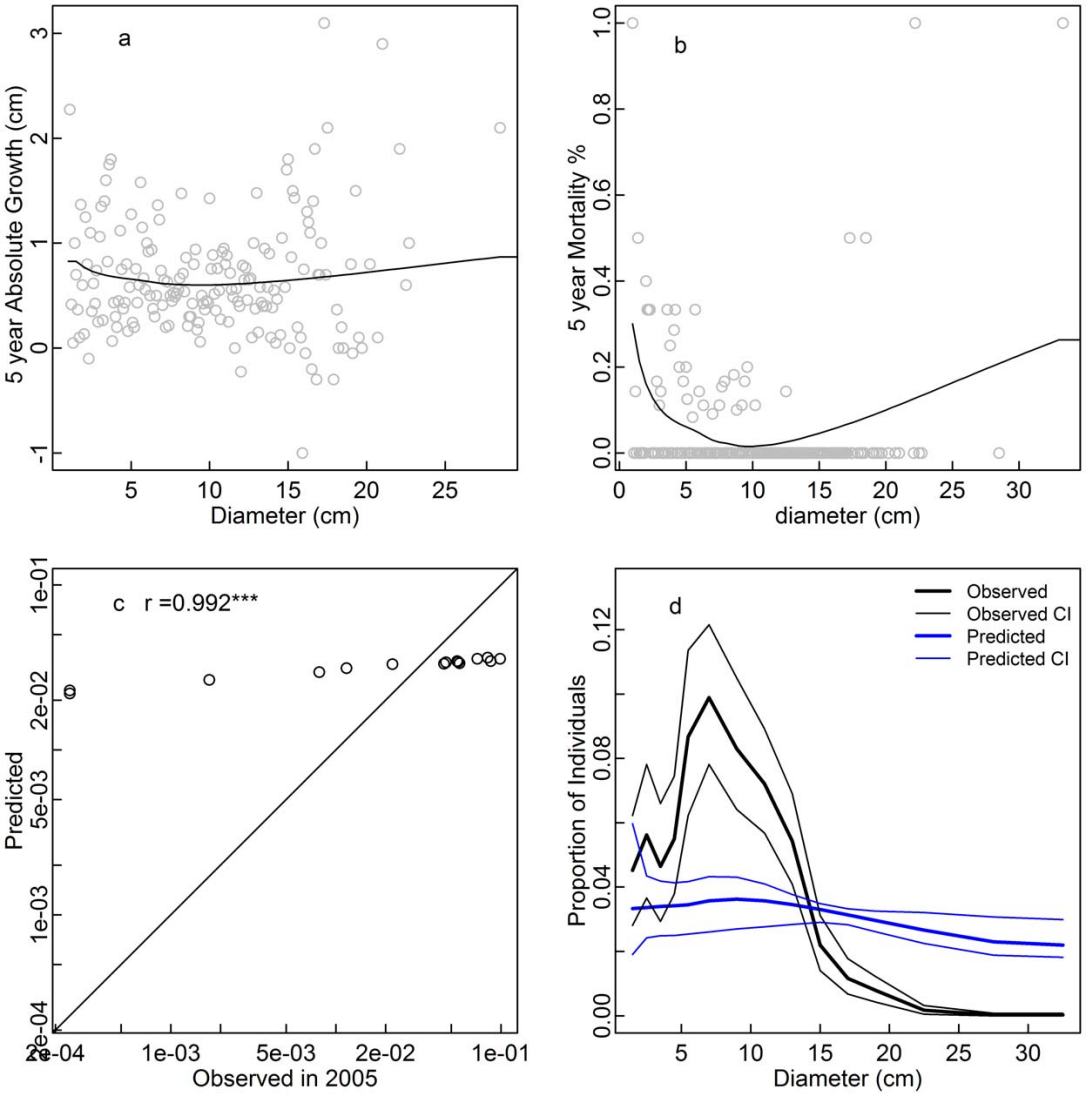
Five year absolute diameter growth (a), five year mortality rate (b), and observed 2005 vs. predicted equilibrium proportions of individuals in each size class (c,d) for *Rapanea neriifolia*. The prediction was derived from growth and mortality functions under the assumption that the population is at demographic equilibrium. Confidence intervals were obtained from bootstrapping over 20×20 m subplots (d).

The quantitative comparisons of observed size distributions with predicted equilibrium size distributions yielded mixed results (Table 1). In 24 of 31 (77.4%) species (18 reversed J and 6 modal), we found strong and significant correlations (Pearson’s r>0.5, p<0.05) between the predicted and observed log-transformed proportions of individuals in different size classes (Table 1). In 12 of 31 species (10 reversed J and 2 modal), predicted and observed confidence intervals on densities overlapped in over 50% of the among size classes (Table 1). *Eurya macartneyi* is one of the species with both high Pearson’s r and high proportion of size classes in which the prediction and observation was not significantly different (Table 1; Figure 3). High proportion of non-significantly different size classes did not necessarily mean high correlations between predicted and observed size distribution (Pearson r = 0.08, p = 0.661). For example, *Rapanea neriifolia* had the largest log-transformed correlation coefficient (Table 1; Pearson’s r = 0.992***, Figure 4 d), but predictions fell within the corresponding bootstrapped confidence intervals for only 33.3% of diameter classes (Table 1; Figure 4 c).

**Figure 5:**
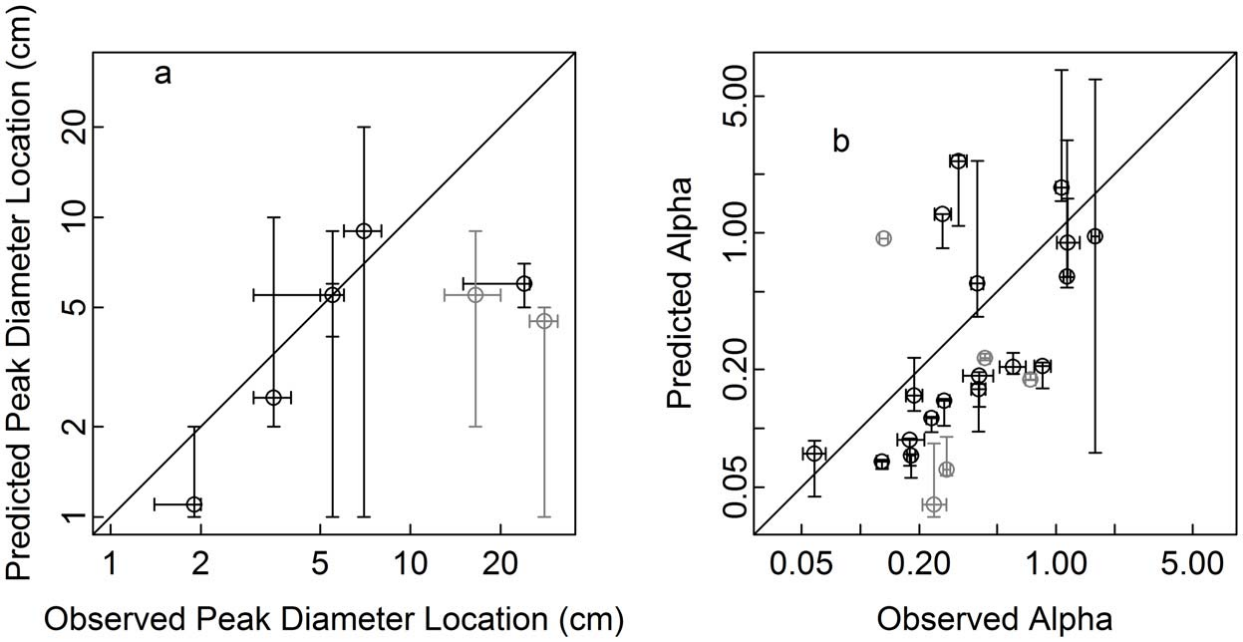
A comparison of the observed size distribution parameters with those predicted from growth and mortality under the assumption of demographic equilibrium: (a) peak locations for modal species and (b) rate parameter estimates of the negative exponential function (eq 1) for reversed J species. 95% confidence intervals obtained by bootstrapping over 20×20 m subplots are shown with bars to allow easy comparison with the 1∶1 line (solid line). Black highlights species with high positive correlations (r>0.5, p<0.05) between observed and predicted tree densities in different size classes, while species with lower correlations are shown in grey. Note that the peaks of the species with a reversed J predicted size distribution were at the smallest size classes.

Among the eight modal species, five had predicted peak locations that were not significantly different from the observed peak locations, while the remaining three had their predicted peak at a significantly smaller diameter than observed (Table 1; Figure 5 a). Observed and predicted peak locations showed positive but non-significant correlated (Spearman correlation = 0.52, p = 0.1911). Among the 23 reversed J species, the predicted rate of decline was not statistically distinguishable from the observed in just six species; in 13 species it was smaller, and in 4 it was larger (Table 1; Figure 5 b). There was a significant positive correlation between the observed and the predicted rate of decline (Spearman r = 0.58, p = 0.004599).

**Figure 6:**
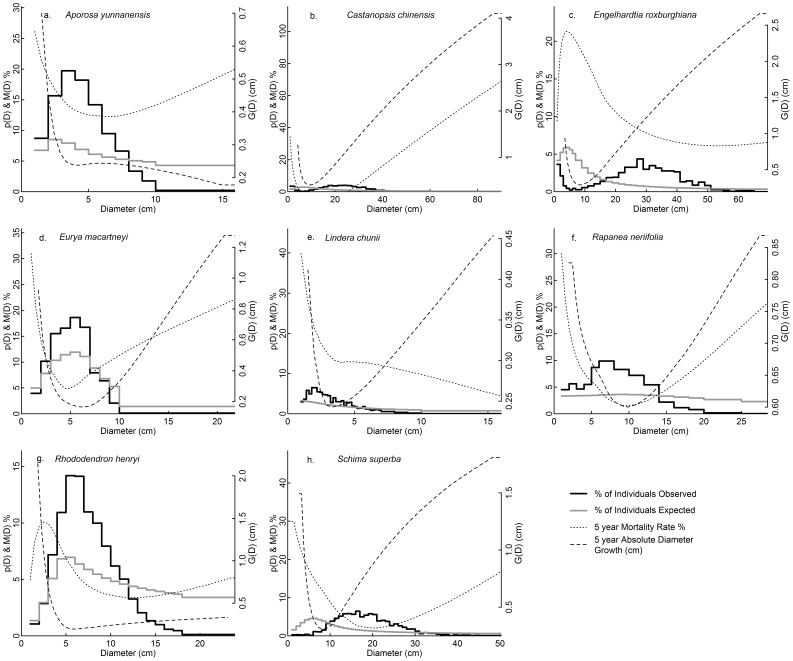
The size distributions in 2005 (p(D)), size distribution equilibrium predicted from empirical motality and growth functions, 5 year mortality rates(M(D)) and absolute diameter growth (g(D)) for the eight modal species. Absolute diameter growth and mortality curves were from loess fits. Corresponding figures for the reversed J species were given in Figure S3.

We found that the observed location of the peak in the size distribution in modal species consistently occurred around the size classes with low mortality and low absolute diameter growth (Figure 6). All modal species had U-or-L shaped mortality functions and absolute growth functions (Figure 6). When the dips were not obvious, the species would have near-reversed J shaped size distributions when at equilibrium (Figure 6 c, f). Dips in mortality and growth curves were also found among reversed J species, but only four reversed J species showed co-occurrences of dips in both mortality and absolute growth functions (Figure S3 d, l, o, q). Three of these species were expected to have a modal size distribution even though they did not have a modal distribution in 2005 (Figure S2), and indeed two of them were already modal in 2010 (Figure S1 c, k).

## Discussion

### Size Distributions and Population Growth

When examining tree size distributions, there is a popular view that a scarcity of juveniles relative to adult should be indicative of population decline [3], [4], [28], [29], [30]. In our study, we found some trends for modal size distribution to be associated with measures of population decline, but these associations were not universal and usually not statistically significant. There was no significant association between size distribution and 5-year population trends among 31 species with 500 or more individuals. Modal species did have significantly lower per capita recruitment rates, but they also had a partially compensating trend towards lower per capita mortality rates, albeit these lower mortality rates of modal species were largely consequences of having fewer small individuals than reversed J species. In all, seven of eight modal species had negative per capita population growth (although in only one case significantly different from zero), compared with 15 of 23 reverse J species.

Though modal species had significantly lower recruitment rates between the two censuses, these short-term recruitment rates may not be a good indicator of how these species are recruiting over the long term. Populations can be divided into two broad categories based on their temporal variation in reproduction: birth flow populations in which reproduction is continual, and birth pulse populations in which reproduction is episodic [31]. Tree populations are likely to lie somewhere between these two ideal extremes. It is possible that the modal species in our study are closer to birth pulse populations. They may recruit mostly in larger occasional pulses with continuous low flow. When the forest is recensused more frequently than the birth pulse takes place, it is easy to erroneously conclude that the population is having difficulty regenerating, when in fact the population is simply recruiting episodically. Episodic recruitment could arise due to mast seeding [32], and/or to temporal variation in regeneration conditions. However, the possibility that the lower recruitment rate in modal species results from episodic seed production is low given that a seed requires a long time to become a 1 cm-DBH recruit, during which the effect of seed pulse are likely to be masked by the variation in growth among individuals.

Other recent studies have also found little support of a link between size distributions and population trends. Condit et al. [5] found that size distribution was not a good indicator of 13-year population trends among 216 tropical tree species with 15 or more individuals each in a 50-ha plot of old-growth tropical forest. Similarly, some study found little association between size distribution and 15-year population trends among 35 species in a 0.89 ha plot in a Japanese temperate fir-hemlock forest [4].

Overall, our results support the view that size distributions are not reliable predictors of population trends. At the same time, we agree with previous finding that size distribution remains a useful, simple tool for identifying species potentially vulnerable to future population declines, albeit a tool that should be used with caution because it is only a weak predictor [4].

### Size Distributions and Life History

Previous studies have also suggested an association between size distribution and shade tolerance in old-growth forests. This association was found among 73 species in a moist tropical forest [2], among four species in a balsam fir-black spruce forest [33], and when species within a light guild were combined and considered together in a mixed-conifer forest [34]. In contrast, in gap-phase stands, shade-intolerant species can also show reversed J size distributions [34]. Here, we failed to find a significant association between shade tolerance and size distribution among 31 species in a subtropical forest, although the trends were consistent with expectations: 80% of shade-tolerant species were reversed-J, as were 74% of intermediate species and just 50% of light-demanding species (Conversely, shade-tolerants constituted 35% of reversed J species vs. 25% of modal, light-demanding species 4% and 13%, and intermediate species 61% and 63%.) and reversed J species have slightly higher continuous shade tolerance, i.e., juvenile survival probability in crowded conditions, than modal species.

The degree of shade tolerance changes with size in many species [35], a reality that is not reflected in simple shade-tolerance classifications and that complicates tests of an association with size distribution. Because the hypothesized link to modality is based on the idea that light-demanding species quickly grow through the smallest size classes, we would expect the strongest association between size distribution and the degree of shade-tolerance of the smallest size classes included in the dataset (here approximately 1–3 cm diameter). For modal species, the forest layer that “the small individuals quickly grow into” can be the canopy, the midstory, or the understory – and in the last case, it may be associated with very high shade-tolerance as large saplings.

In our study, modal species were significantly associated with a particular life history pattern, albeit not the light-demanding strategy. All modal species had strongly U or L-shaped relationships of growth and mortality with diameter, with at least one of the two being U-shaped. Although U-shaped mortality curves are common in both reversed J and modal species, the depth of the dip in modal species is much more pronounced. The deeper dip in mortality curves of modal species led to relatively higher mortality of the small stems and lower mortality of intermediate stems where slow diameter growth is found. Thus the stems of modal species accumulate at intermediate sizes. In addition, in all these species, the slow-down in diameter growth begins occurs at approximately the size where reproduction commences (Cao, personal communication). The simultaneous dip in mortality suggests that these species have a fairly conservative strategy at these intermediate sizes; we speculate that they are allocating strongly to survival-enhancing traits, moderately to reproduction, and little to growth at these sizes, and thus are able to survive for long periods even at low resources.

### Comparing Observed and Equilibrium Size Distributions

Comparisons of observed size distributions with projected equilibrium size distributions provide insight into whether populations – and the forest – have reached equilibrium. We found considerable mismatches between observed and predicted equilibrium size distributions among our focal species, providing evidence that this forest is undergoing directional change. In 19 of 31 species, more than half the size classes exhibited significant differences between observed and predicted proportions. Only two species showed no significant differences in any size class. At the same time, predicted and observed proportional allocation among size classes were significantly positively correlated in 25 of 31 species, suggesting that current size-dependent growth and mortality patterns were reasonably similar to the past patterns that shaped the current size distribution.

The differences in the exponential rates of decline between observed and projected equilibrium distributions in the reversed J species suggest that many species have experienced systematic changes in the size-dependence of growth and/or mortality over time. Seventeen of 23 reversed J species exhibited a trend towards faster rates of decline in observed size distributions than in projected equilibrium distributions, and 13 of these species has significantly greater rates of decline. Such patterns could result if in the past small individuals of these species had relatively higher growth and/or relatively higher survival than they do in the present – perhaps because of greater resource availability to smaller individuals in the past. That in turn is consistent with ongoing succession. In contrast, four reversed J species exhibited significantly slower rates of decline in observed size distributions than in projected equilibrium distributions – perhaps because of decreases in recruitment over time. In general, differences between observed and projected equilibrium distributions could also reflect responses to long-term temporal climatic variation, whether directional or cyclical [36].

We used multiple approaches to compare observed with projected equilibrium size distributions. Previously, size distributions were compared using visual inspection [15], [19] and parameter confidence intervals [10]. We compared visual classifications of the shape of size distributions, estimated parameters, and also proportions of individuals in size classes. For the numerical measures, we evaluated the match in terms of the correlation between observed and predicted, as well as by evaluating overlap of confidence intervals. We generated confidence intervals on both observed and projected size distributions and their parameter estimates based on bootstrapping over subplots. These approaches in combination provided stronger tests of the agreement – or lack thereof – between observed and predicted equilibrium size distributions. In particular, they highlighted a preponderance of similarities in type of shape combined with significant numerical differences in shape parameters and in the proportions of individuals in different size classes.

We projected equilibrium size distributions expected if observed recruitment and observed size-dependent patterns of growth and mortality remained constant into the future. This approach follows in a long tradition of work based on the one-dimensional continuity equation of fluid dynamics with a mortality term (reviewed by 16). We used locally weighted regression to flexibly fit growth and mortality as a function of size. As a result, the complex growth and mortality patterns were fully taken into account in the size distribution projections, which had to be made numerically. This contrasts with the approach of Muller-Landau et al. [37], who fitted power functions for growth and mortality. These power functions did not capture the full complexity of the growth and mortality patterns, but did enable analytical solution of the size distribution and a clear identification of the links of the size distribution parameters with the growth and mortality parameters. More complex models for growth and mortality can include influences of stand structure, such that growth slows and mortality increases as competition increases over the course of succession. Such approaches are more useful for modeling the temporal evolution of size distributions over the course of succession. Because these models are more complex, they require more data for accurate parameterization, and also can be solved only numerically, i.e. by simulation [17], [18], [19], [20].

Our results suggest that succession proceeds slowly and over a very long time in this forest. Following Condit et al [38], we fit seedling growth rates (from an independent 3-year dataset) as polynomial functions of log-transformed DBH, and then integrated the resulting differential equation of DBH to obtain age for a given size. These calculations suggest that it takes 101 years before a seedling reaches a height of 144 cm. By comparison, a 1 cm DBH recruit into the main census has an average height of 2.33 m (data from a 1-ha plot adjacent to the 20-ha plot). The average seedling at our plot thus grows more slowly than balsam fir (*Abies balsamea*), black spruce (*Picea mariana*) and white spruce (*Picea glauca*), which required 62, 40, and 48 years, respectively, to reach 1.3 m [34]. Given these rates, it is not surprising that even this very old forest appears still to be in succession.

### Conclusions and Recommendations

Fundamentally, two processes affect tree size distributions: life history as reflected in size-dependent changes in growth and mortality and temporal variation in recruitment. Thus size distributions are not reliable predictors of either life history or population trends alone. Previous studies have found that modal size distributions may be associated with either juvenile shade-intolerance, or declining populations. Here, we show that modal distributions in a subtropical forest are not explained by either of these factors, but instead primarily reflect a life history characterized by an intermediate size class with markedly lower growth and mortality.

We used the one-dimensional continuity equation of fluid dynamics to predict the equilibrium size distribution. The precise allocations of individuals among size classes deviated markedly from the observed in two thirds of the species. The predictions on the rate of decline in reversed J species were generally smaller than the observed. These results indicate that this forest is undergoing ongoing change, most likely due largely to late succession, although environmental change be also be involved. Future research should evaluate changes in growth and mortality over time, to establish the degree to which these are changing directionally and/or whether they are related to interannual variation in climate. In addition, it would be useful to analyze the dependence of neighborhood competition, and thereby build a solid basis for short-term and long-term projection of the temporal evolution of individual species size distributions as forest change proceeds.

## Supporting Information

Figure S1 **Size distributions of the remaining reversed J species that were not shown in** **Figure 1** **.** Five species have non-significant peaks outside the smallest size class in 2005 (f, h, k, s, t).
(DOC)

Figure S2 **Growth (a), mortality (b) functions and comparisons of the observed sized distributions in 2005 and the expected equilibrium size distributions (c, d) for all the studied species.**
(DOC)

Figure S3 **The size distributions in 2005 (p(D)) (black solid lines), 5-year mortality rates(M(D)) (dotted lines), 5-year absolute diameter growth rates(g(D)) (dashed lines), and size distributions expected based on growth and mortality (grey solid lines) for the reversed J species.** Absolute diameter growth and annual mortality curves were from loess fits.
(DOC)
