# Supplementary material for: Unimodal Tree Size Distributions Possibly Result from Relatively Strong Conservatism in Intermediate Size Classes
Source: PLoS One. 2012 Dec 31;7(12):e52596. doi: 10.1371/journal.pone.0052596 (PMC3534107; doi:10.1371/journal.pone.0052596)
Supplement: Figure S1 — Size distributions of the remaining reversed J species that were not shown in Figure 1 . Five species have non-significant peaks outside the smallest size class in 2005 (f, h, k, s, t). (DOC) [file pone.0052596.s001.doc]

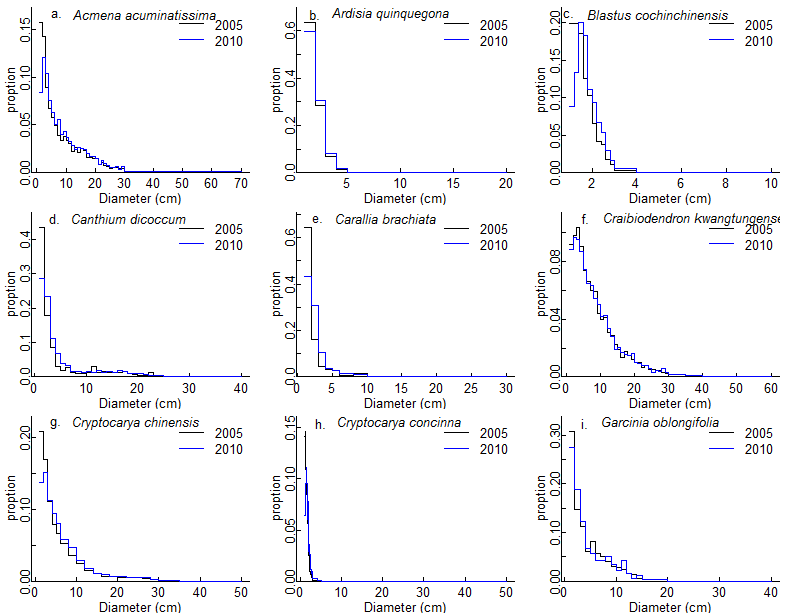


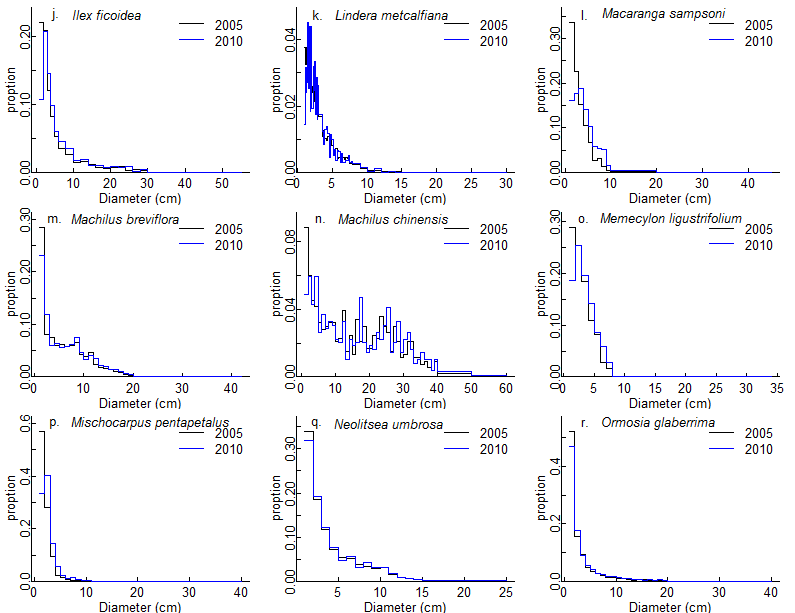


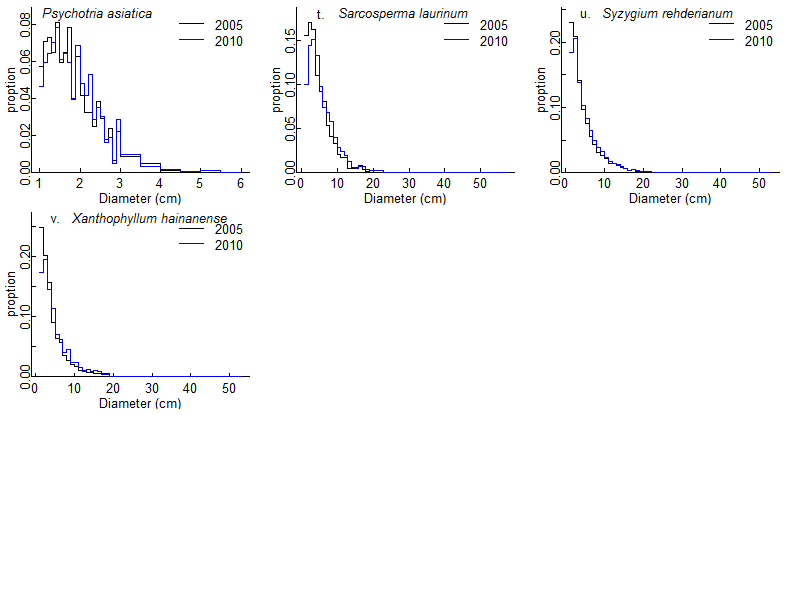


Figure S1. Size distributions of the remaining reversed J species that were not shown in Figure 1.Five species have non-significant peaks outside the smallest size class in 2005 (f, h, k, s, t).
