# Supplementary material for: Unimodal Tree Size Distributions Possibly Result from Relatively Strong Conservatism in Intermediate Size Classes
Source: PLoS One. 2012 Dec 31;7(12):e52596. doi: 10.1371/journal.pone.0052596 (PMC3534107; doi:10.1371/journal.pone.0052596)
Supplement: Figure S2 — Growth (a), mortality (b) functions and comparisons of the observed sized distributions in 2005 and the expected equilibrium size distributions (c, d) for all the studied species. (DOC) [file pone.0052596.s002.doc]

*Acmena acuminatissim:*


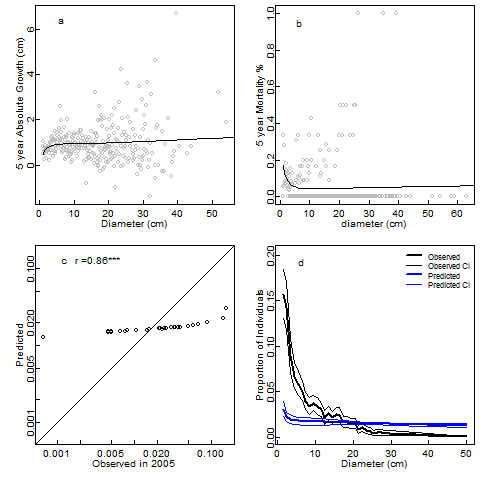


*Aidia canthioides*:


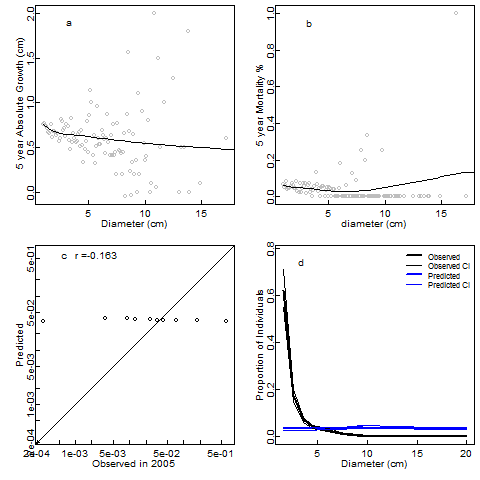


*Aporosa yunnanensis*:


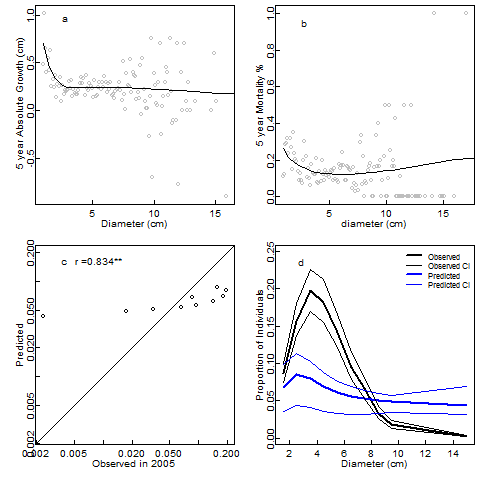


*Ardisia quinquegona*:


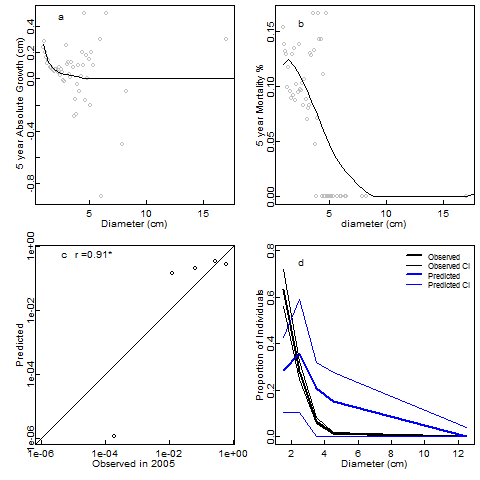


*Blastus cochinchinensis*:


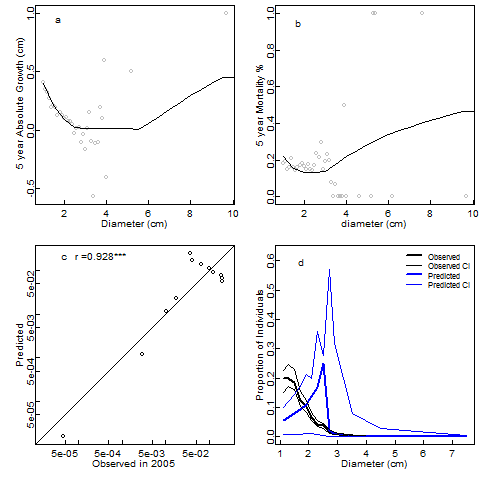


*Canthium dicoccum*:


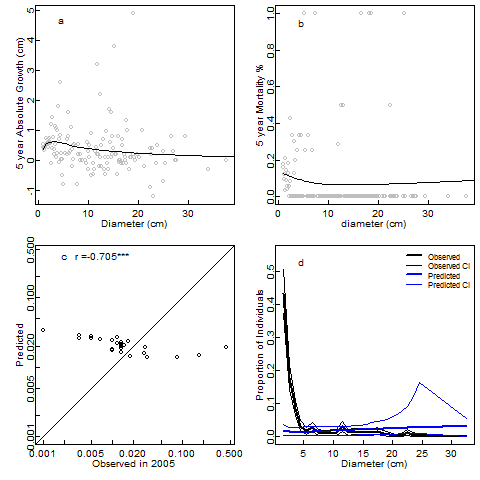


*Carallia brachiata*:


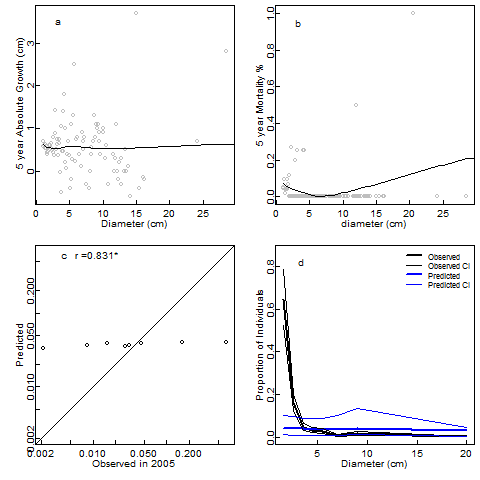


*Castanopsis chinensis*:


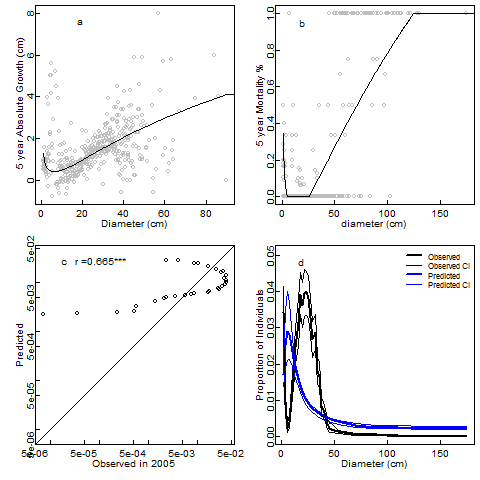


*Craibiodendron kwangtungense*:


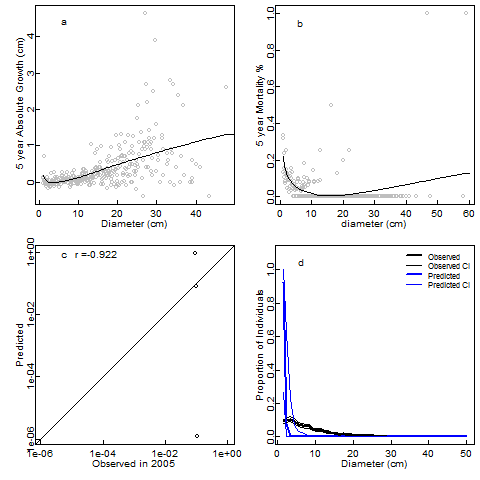


*Cryptocarya chinensis*:


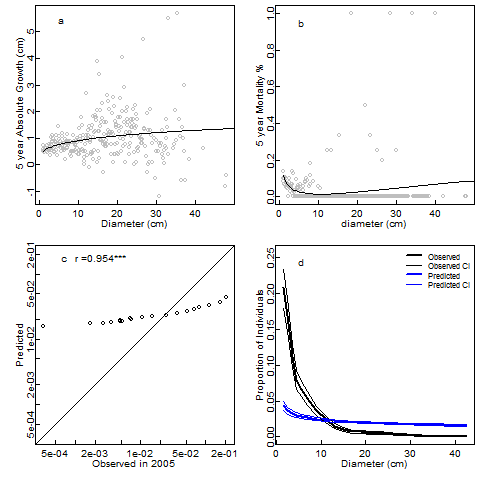


*Cryptocarya concinna*:


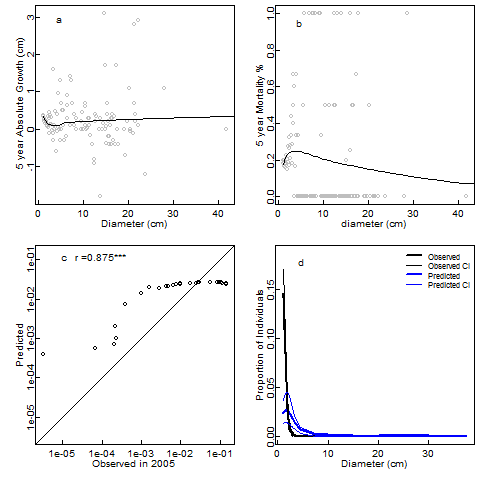


*Engelhardtia roxburghiana*:


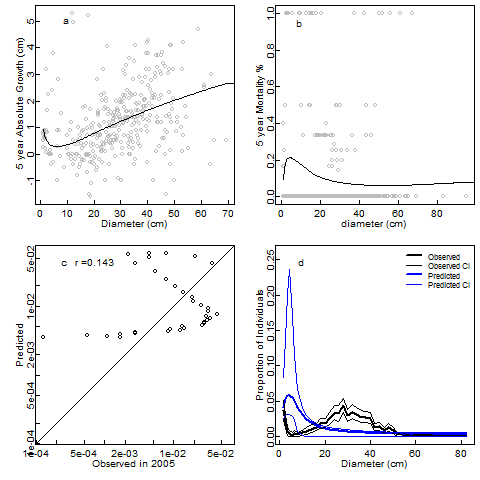


*Eurya macartneyi*:


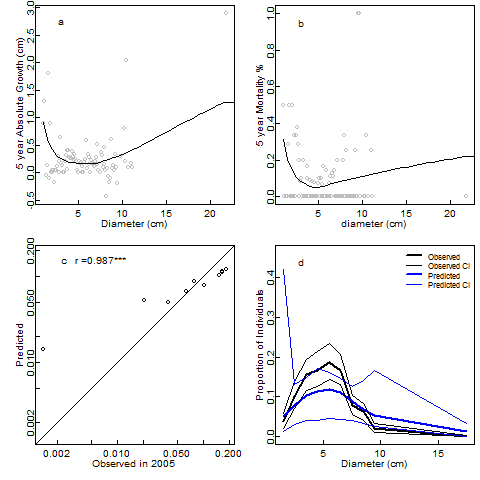


*Garcinia oblongifolia*:


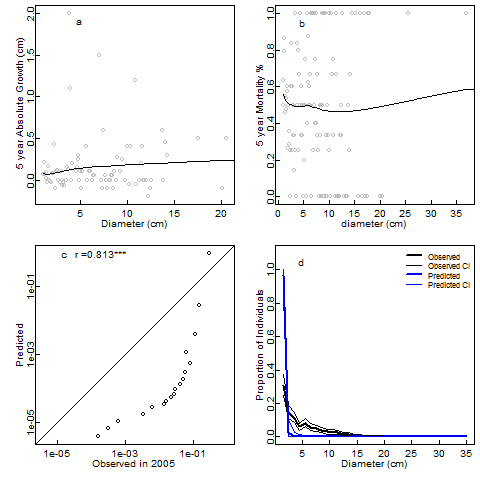


*Ilex ficoidea*:


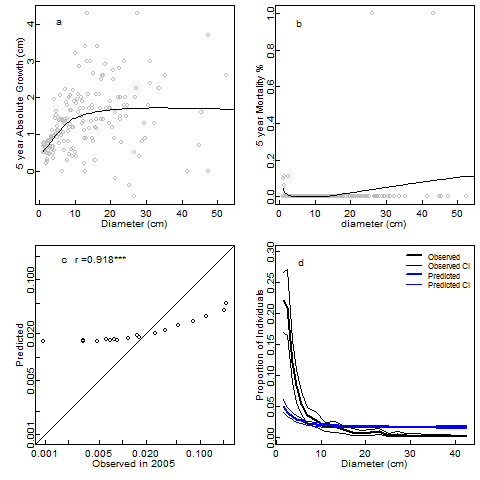


*Lindera chunii*:


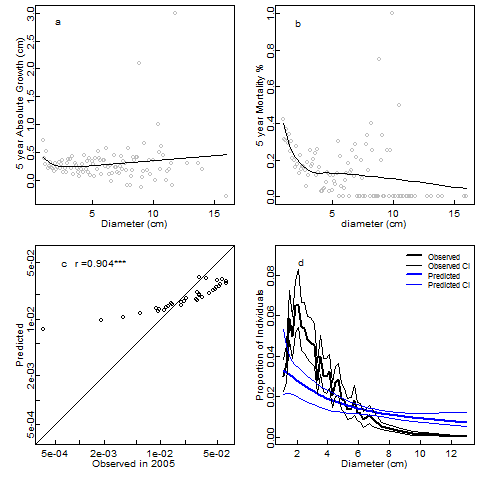


*Lindera metcalfiana*:


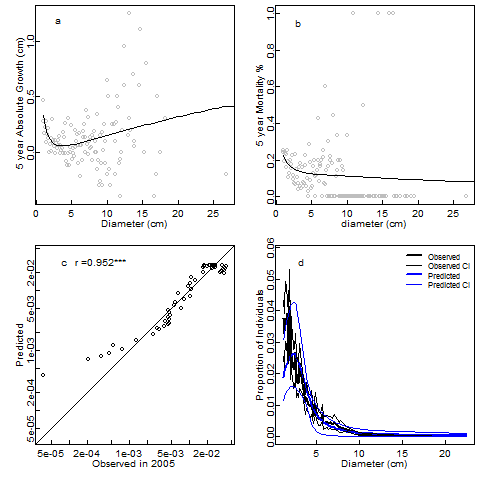


*Macaranga sampsoni*:


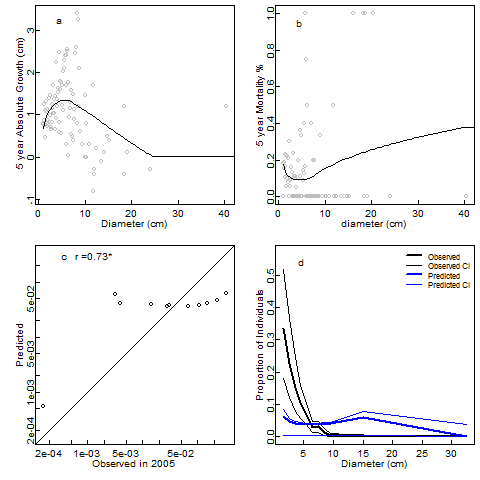


*Machilus breviflora*:


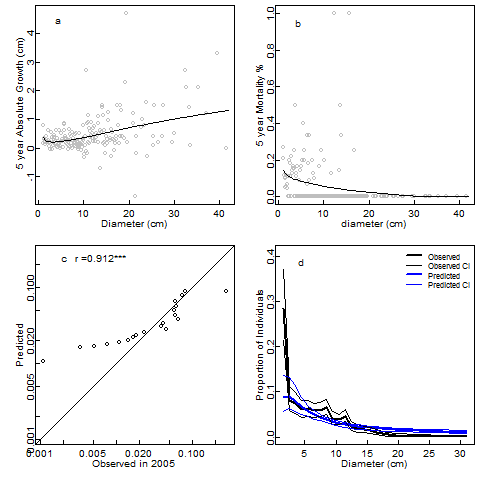


*Machilus chinensis*:


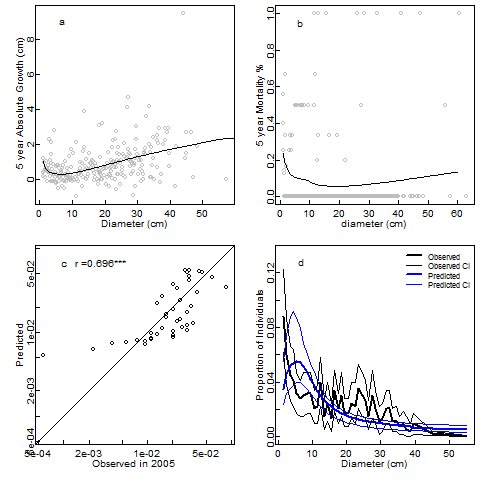


*Memecylon ligustrifolium*:


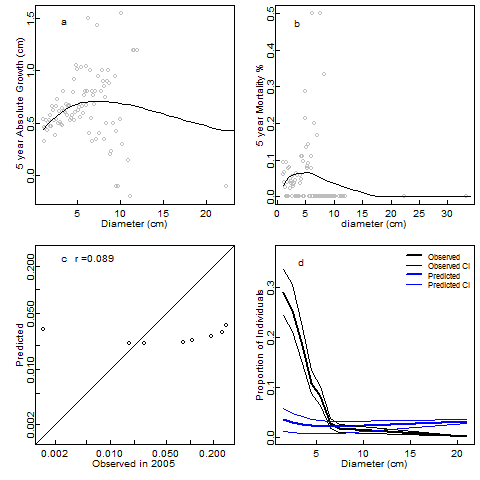


*Mischocarpus pentapetalus*:


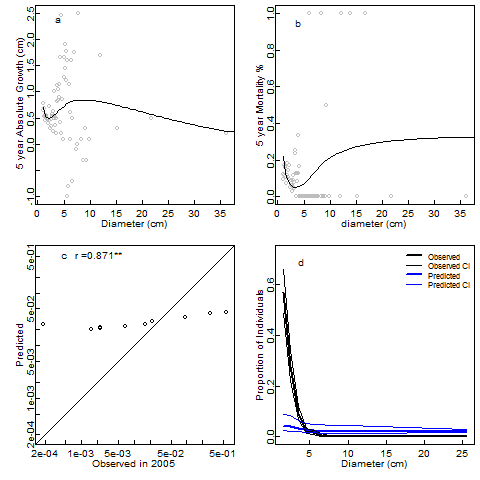


*Neolitsea umbrosa*:


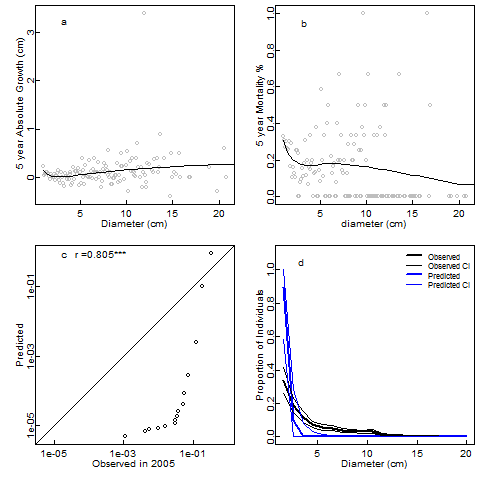


*Ormosia glaberrima*:


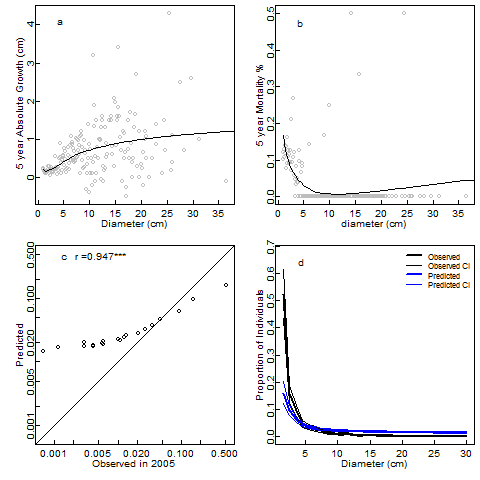


*Psychotria asiatica*:


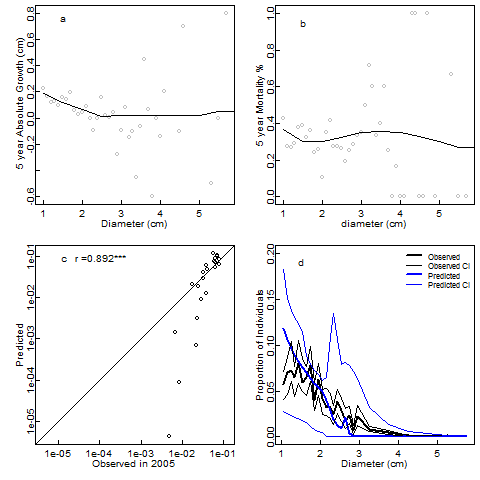


*Rapanea neriifolia*:


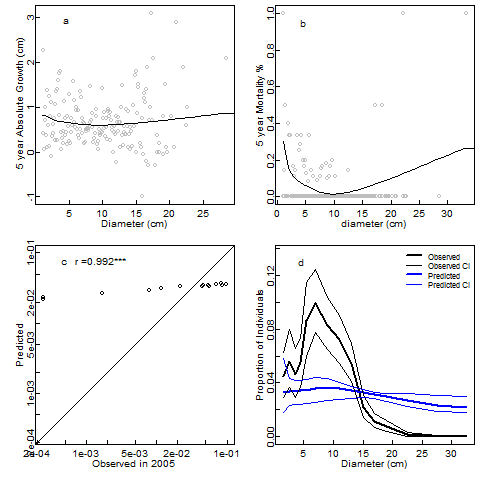


*Rhododendron henryi*:


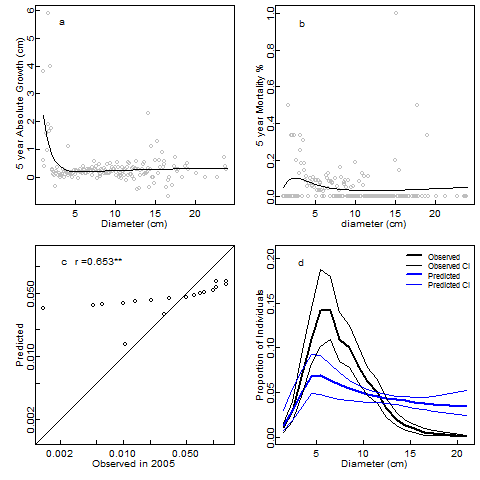


*Sarcosperma laurinum*:


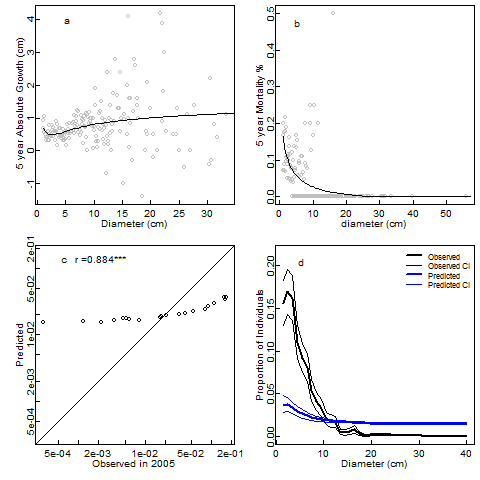


*Schima superba*:


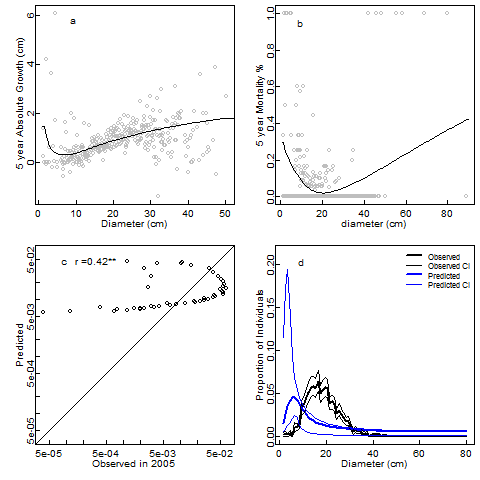


*Syzygium rehderianum*:


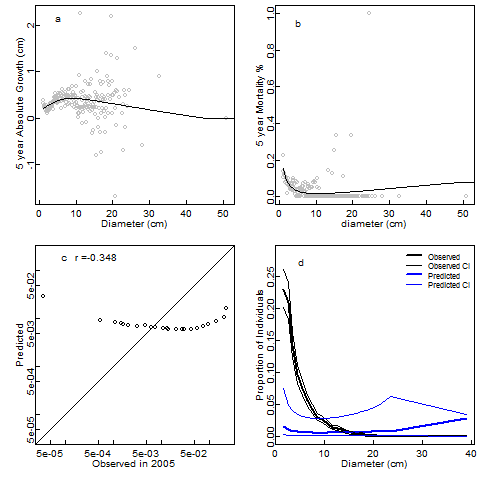


*Xanthophyllum hainanense*:


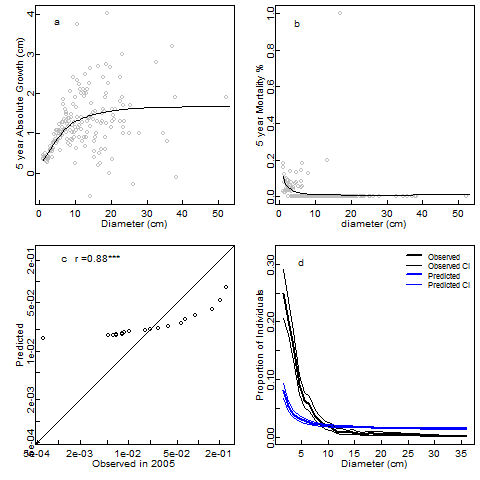


Figure S2. Growth (a), mortality (b) functions and comparisons of the observed sized distributions in 2005 and the expected equilibrium size distributions (c, d) for all the studied species.
