# Supplementary material for: Unimodal Tree Size Distributions Possibly Result from Relatively Strong Conservatism in Intermediate Size Classes
Source: PLoS One. 2012 Dec 31;7(12):e52596. doi: 10.1371/journal.pone.0052596 (PMC3534107; doi:10.1371/journal.pone.0052596)
Supplement: Figure S3 — The size distributions in 2005 (p(D)) (black solid lines), 5-year mortality rates(M(D)) (dotted lines), 5-year absolute diameter growth rates(g(D)) (dashed lines), and size distributions expected based on growth and mortality (grey solid lines) for the reversed J species. Absolute diameter growth and annual mortality curves were from loess fits. (DOC) [file pone.0052596.s003.doc]

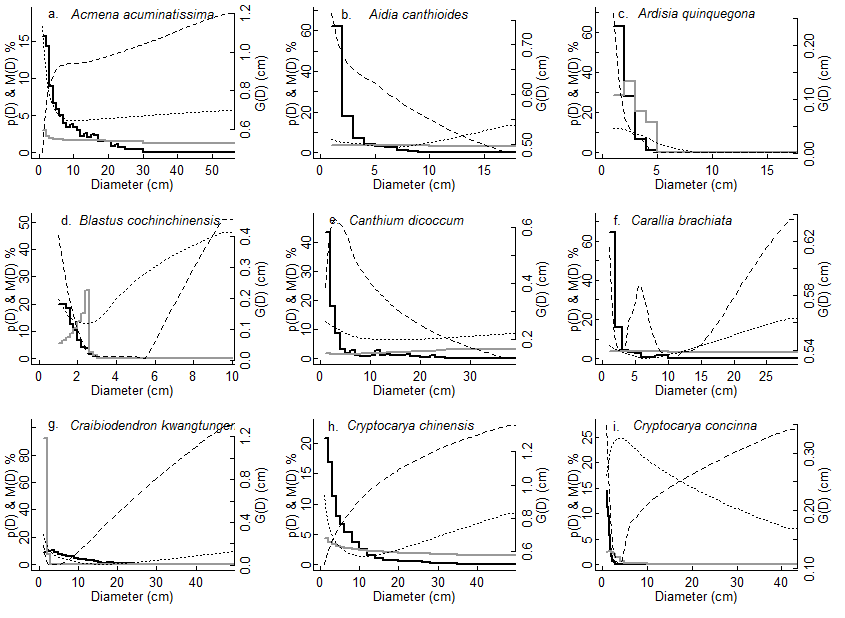

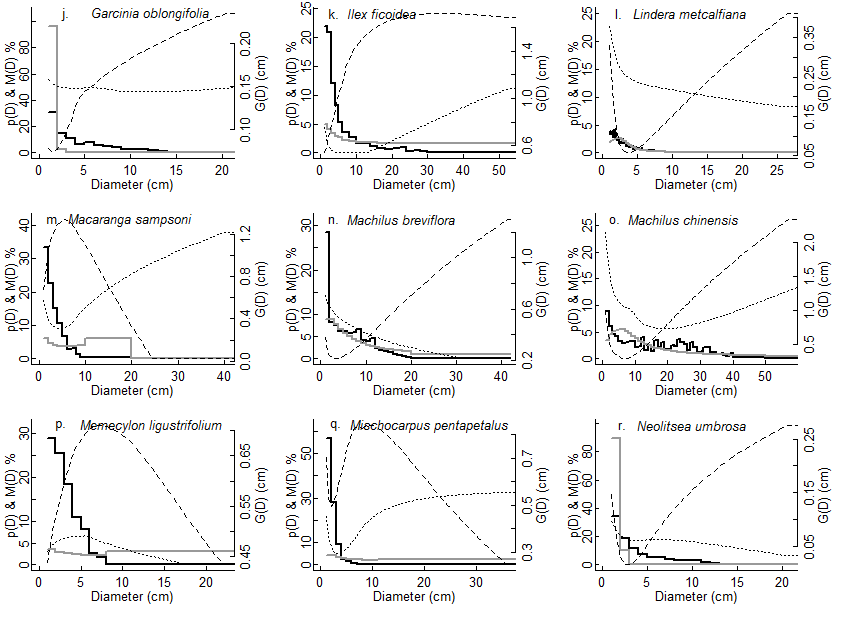


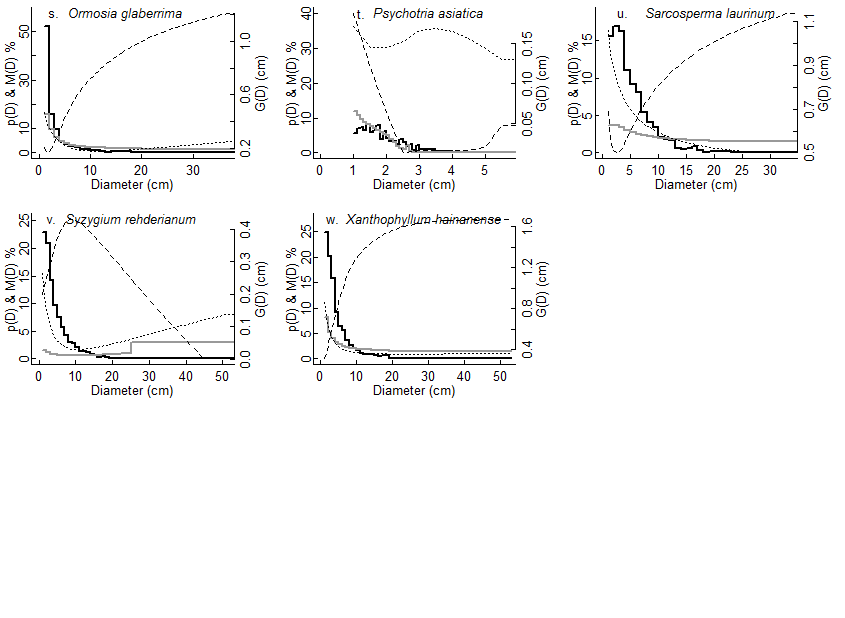


Figure S3. The size distributions in 2005 (p(D)) (black solid lines), 5-year mortality rates(M(D)) (dotted lines), 5-year absolute diameter growth rates(g(D)) (dashed lines), and size distributions expected based on growth and mortality (grey solid lines) for the reversed J species. Absolute diameter growth and annual mortality curves were from loess fits.
